# Cardiorespiratory fitness and the incidence of surgery for aortic valve stenosis—the HUNT study

**DOI:** 10.1093/ejcts/ezad322

**Published:** 2023-09-19

**Authors:** Benedikte Therese Smenes Nystøyl, Jon Magne Letnes, Bjarne Martens Nes, Katrine Hordnes Slagsvold, Ulrik Wisløff, Alexander Wahba

**Affiliations:** Department of Circulation and Medical Imaging, Norwegian University of Science and Technology, NTNU, Trondheim, Norway; Department of Circulation and Medical Imaging, Norwegian University of Science and Technology, NTNU, Trondheim, Norway; Clinic of Cardiology, St Olavs University Hospital, Trondheim, Norway; Department of Circulation and Medical Imaging, Norwegian University of Science and Technology, NTNU, Trondheim, Norway; Department of Circulation and Medical Imaging, Norwegian University of Science and Technology, NTNU, Trondheim, Norway; Clinic of Cardiothoracic Surgery, St. Olavs University Hospital, Trondheim, Norway; Department of Circulation and Medical Imaging, Norwegian University of Science and Technology, NTNU, Trondheim, Norway; School of Human Movement and Nutrition Science, University of Queensland, Queensland, Australia; Department of Circulation and Medical Imaging, Norwegian University of Science and Technology, NTNU, Trondheim, Norway; Clinic of Cardiothoracic Surgery, St. Olavs University Hospital, Trondheim, Norway

**Keywords:** Aortic valve stenosis, Cardiorespiratory fitness, Primary prevention

## Abstract

**OBJECTIVES:**

Aortic valve stenosis (AVS) shares many risk factors with coronary disease, the latter being strongly and inversely associated with physical activity (PA) and cardiorespiratory fitness (CRF). However, the relationship between PA, CRF and AVS needs to be established. We explored whether PA habits and estimated CRF affect the risk of developing AVS demanding aortic valve replacement (AVR) and how these factors affect postoperative mortality.

**METHODS:**

Participants from the second and third waves of Trøndelag Health Study were cross-linked with a local heart surgery registry and the Norwegian Cause of Death Registry. Estimated CRF was calculated through a developed algorithm based on clinical and self-reported data. Fine-Gray competing risk analyses were used to investigate how PA habits and estimated CRF were associated with the risk of AVR across CRF quintiles, PA groups and per 1-metabolic equivalent task (MET) (3.5 ml/min/kg).

**RESULTS:**

In a study population of 57 214 participants, we found a 15% [95% confidence interval (CI) 1–27] reduced risk of AVR per 1-MET estimated CRF increment. Those in the highest CRF quintile had a 56% (95% CI 14–77) lower risk of surgery compared to the lowest quintile. Analyses on PA groups did not show significant results. Finally, we found a 37% (95% CI 17–53) lower risk of postoperative mortality per 1-MET increased estimated CRF.

**CONCLUSIONS:**

Our findings indicate a strong and inverse relationship between estimated CRF and incidence of AVR due to AVS. Higher estimated CRF was associated with lower mortality after surgery.

**Figure:**
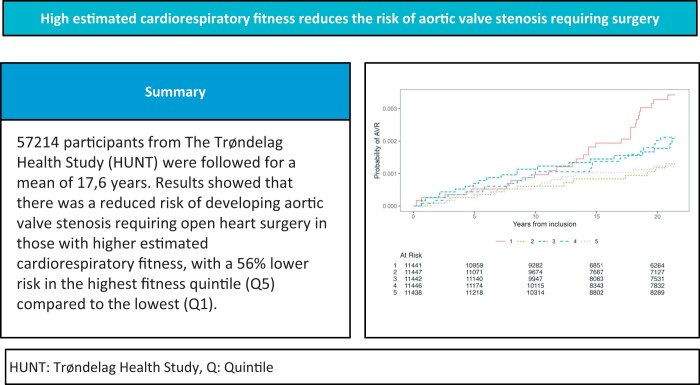

## INTRODUCTION

Despite the dramatic reduction of rheumatic valve disease in industrial countries, the prevalence of valvular surgery has not been reduced correspondingly, due to increasing incidence of degenerative valvular disease [1]. Aortic valve stenosis (AVS) is the most common valvulopathy in adults in the Western world, with an increasing prevalence due to an ageing population [2, 3]. Degenerative AVS shares many risk factors with atherosclerosis and coronary artery disease (CAD); e.g., age, male sex, smoking, diabetes, hypertension and hypercholesterolaemia [4, 5]. The pathophysiological processes also bear similarities, such as endothelial disruption, accumulation of macrophages and lipoproteins, cytokine activation and calcification [6], and is now considered to be more than just age-related wear and tear of the valve [4]. However, all aspects of the pathogenesis in AVS are not fully understood. If untreated, AVS is fatal within a few years after symptom onset, and aortic valve replacement (AVR) remains the only treatment option to date [7]. Prosthetic valves impose new risks such as deterioration of prosthetic biological valves or complications associated with lifelong anticoagulation after implanting mechanical valves [8]. As there currently exists no effective medical treatment options to halt or slow down the progression of AVS, primary prevention of AVS is warranted. Low levels of physical activity (PA) and low cardiorespiratory fitness (CRF) are important risk factors in the development of CAD [9, 10] and one could also expect these factors to be important in AVS. However, the effects of PA and CRF on AVS are scarcely explored.

In a recent study, we found that high levels of PA and high estimated cardiorespiratory fitness (eCRF) were inversely associated with the incidence of CAD demanding coronary artery bypass grafting (CABG) surgery [11]. We therefore wanted to explore whether the same interrelationship could be found for AVS requiring AVR. This study aimed to explore the association between PA and eCRF, respectively, and the incidence of severe AVS requiring AVR in a healthy population. AVR was chosen as this represents documented severe AVS and is a clear end point of AVS. Further, we wanted to identify risk factors for undergoing heart surgery in particular. Moreover, we wanted to examine how PA and eCRF were associated with mortality after AVR. Our hypotheses were that high PA and eCRF are inversely associated with the incidence of AVR surgery and mortality after surgery.

## PATIENTS AND METHODS

### Ethics statement

The Regional Committee for Medical Research Ethics Northern Norway approved the study (REK 2018/986). All participants gave informed and written consent before participating in the Trøndelag Health Study (HUNT).

### Study design and population

The Trøndelag Health Study (HUNT) is a large population cohort based on the adult population of Trøndelag County in Norway, containing medical and health-related data. The study has been performed in 4 waves (HUNT1–HUNT4) since 1984. HUNT2 was carried out between 1995–1997, where 69.5% (*n* = 65 237) of the population participated. HUNT3 was undertaken in 2006–2008, with 54.1% responders (*n* = 50 807). Inclusion involved questionnaires regarding health and lifestyle, and a clinical examination. Study details can be found elsewhere [12]. This study included 57 214 participants from HUNT2 and HUNT3, preferably HUNT2 if subjects had participated in both. Participants with a history of angina pectoris and/or myocardial infarction were excluded, as well as those who had undergone heart surgery prior to the baseline examination.

### Questionnaire-based and clinical variables

We obtained self-reported data from the HUNT2 and HUNT3 questionnaires. Cigarette smoking was categorized as ‘yes’ if participants were current or occasional smokers and ‘no’ if they reported to be never or ex-smokers. Alcohol consumption was classified based on the number of units per 2 weeks: Abstainer (0), 1–4 drinks or ≥ 5 drinks. Occupational status was divided into 6 groups: higher-grade professionals, lower-grade professionals, routine nonmanual workers, manual workers, non-workers and retired. Patients were classified as having a positive family history of CAD if they had a first-degree relative with a myocardial infarction before the age of 60. Clinical variables included resting heart rate (rHR), waist circumference, and systolic and diastolic blood pressure, which was measured by trained nurses upon inclusion. Blood pressure and rHR were measured 3 times with 1-min intervals with the use of a Dinamap 845XT (Critikon, Tampa, USA), using the lowest measurement of the 3 for rHR and the mean of the second and third measurement for blood pressure. Waist circumference was measured at the umbilicus level and reported to the nearest 1 cm [13].

### Assessment of physical activity

HUNT2 and HUNT3 included 2 previously validated questions regarding PA [14]. Participants were requested to report the volume of light intensity and high-intensity PA per week with the alternatives ‘none’, ‘<30 min’, ‘1–2 h’ and ‘3 h or more’. Based on this, participants were categorized into: Less active (<1 h hard and <3 h light PA per week), moderate active (1–3 h hard or 3 h light PA per week) or active (>3 h hard PA per week), based on a previously published classification [15]. Self-reported PA was also used to calculate eCRF, based on a previously published non-exercise model established from ∼4500 participants from HUNT3 undergoing cardiopulmonary exercise testing as part of The HUNT3 Fitness Study [16]. The model is sex-specific and based on whether participants meet the recommendations of PA (≥75 min vigorous PA or ≥150 min moderate PA, or a combination of the 2), age, waist circumference and rHR.

### Ascertainment of outcomes

Primary outcome of this study was incident isolated AVR surgery due to high-grade AVS (mean gradient ≥40 mmHg), excluding transcatheter aortic valve implantation (TAVI) procedures. The secondary outcome was mortality after AVR surgery. Data from the HUNT was cross-linked with the heart surgery register at St. Olavs University Hospital, Trondheim, Norway. The latter is an extensive collection of anamnestic and clinical data regarding all patients undergoing open-heart surgery at St. Olavs Hospital, the only hospital with cardiac surgery serving the inhabitants of Trøndelag County. Cross-linking of these 2 registries would therefore identify nearly every HUNT participant undergoing AVR surgery. AVS was defined as having an echocardiographic mean gradient ≥40 mmHg over the aortic valve. This cut-off was chosen to be sure that the primary indication for surgery was AVS and not for instance aortic insufficiency or CAD. Those with low flow/low gradient AVS were not easy to identify as detailed echocardiographic measures were not included in the heart surgery database. Further, data were cross-linked with the Norwegian Cause of Death Registry (NcoDR) to evaluate postoperative mortality.

### Statistical procedures

Descriptive data are presented with means and standard deviations (SDs) for continuous variables and numbers with percentages (%) for categorical variables. All-cause mortality and cardiac surgery other than isolated AVR due to AVS were defined as competing risks since this could affect the primary indication for AVR. This included for instance AVR due to aortic valve regurgitation, TAVI and infective endocarditis. Cumulative incidence of isolated AVR, other cardiac surgical procedures and death are presented graphically by levels of PA and eCRF. End of follow-up was December 2017. An extension of the Fine-Gray competing risk regression method for stratified data was used to account for competing risks of undergoing isolated AVR due to AVS. This allows the baseline hazard function to differ across different strata of 10-year age groups [17]. The results are presented as hazard ratios with 95% confidence intervals (CIs) and are based on separate models for the predictors of interest (PA and eCRF). We constructed 2 models to adjust for confounding factors in the relationship between PA/eCRF and outcomes. Model 1 was adjusted for sex and age upon inclusion, whereas model 2 was further adjusted for smoking, alcohol consumption, family history of CAD and occupational status. In sensitivity analyses, we repeated analyses with a composite end point of all AVR surgery due to AVS, meaning that surgeries with concomitant procedures such as CABG were included. eCRF was further divided into sex-specific quintiles within each 10-year age group (<40, 40–49, 50–59, 60–69, ≥70) before combining them [18]. To allow for flexible modelling of the eCRF predictor a model using restricted cubic splines in a Cox proportional hazard model, censoring other surgical procedures at the time of surgery was also fitted. Testing for non-linearity was performed by a likelihood ratio test comparing models with restricted cubic splines term of 2–5 knots for eCRF with a linear model. A Cox proportional hazards model was used to assess postoperative mortality, where follow-up time was defined as time from surgery to death or end of follow-up in March 2020. We constructed 2 models similar to those in the incidence analyses, but also adjusted for time between participation in HUNT and surgery in both models. Non-linearity of the eCRF predictor was tested comparing a model using restricted cubic splines towards the model using a linear term for eCRF. All other covariates were collected from the HUNT baseline examination. Body mass index was not adjusted for, as waist circumference was part of the eCRF algorithm, which could potentially lead to collinearity and overadjustment. We also analysed the risk of AVR based on rHR and waist circumference to look more closely at the components of eCRF.

The proportional hazards assumption was assessed by testing Schoenfeld residuals. Hazard ratios are reported according to PA categories, per metabolic equivalent task (MET ∼ 3.5 ml/min/kg) of eCRF and by eCRF quintiles. Analyses were performed using SPSS for Macintosh version 26.0 (IBM_SPSS Inc., Armonk, NY, USA) and R 4.05 (www.r-project.org, Survival [version 3.2–10], cmprsk [version 2.2–10] and crrSC [version 1.1] packages) (R Foundation for Statistical Computing, Institute for Statistics and Mathematics, Vienna, Austria).

## RESULTS

We followed a total of 57 214 participants from HUNT2 or HUNT3 for a mean follow-up time of 17.6 years (1 005 670 person-years). 102 participants underwent AVR due to AVS during follow-up, giving a population incidence of 0.10 per 1000 person-years. The mean age at inclusion was 45.9 (SD 16.0), and the mean age at AVR surgery was 70.1 (SD 9.8) years. Baseline characteristics are outlined in Table 1.

**Table 1:** Baseline characteristics of study population, stratified by sex

	Men	Women
	*n* = 26 704	*n* = 30 070
Age (years)	46.1 (15.7)	45.7 (16.3)
eCRF (ml/min/kg)	46.0 (8.1)	36.3 (7.1)
Waist circumference (cm)	92.0 (9.7)	82.1 (12.1)
Body mass index	26.5 (3.5)	26.0 (4.6)
Systolic BP (mmHg)	137 (18)	130 (21)
Diastolic BP (mmHg)	80 (12)	76 (12)
Resting heart rate (beats/min)	68 (12)	71 (12)
Total cholesterol (mmol/l)	5.7 (1.2)	5.7 (1.3)
HDL cholesterol (mmol/l)	1.2 (0.3)	1.5 (0.4)
Triglycerides (mmol/l)	1.9 (1.3)	1.4 (0.9)
Current smoker (%)	27.1	29.0
Family history CVD (%)	15.2	17.4
Alcohol units last 2 weeks	5.0 (6.3)	2.5 (3.6)
Isolated AVR	39	63
AVR incl. concomitant procedures	110	93

Values are mean (SD), % or *n*.

AVR: aortic valve replacement; BP: blood pressure; CVD: cardiovascular disease; eCRF: estimated cardiorespiratory fitness; HDL: high-density lipoprotein; SD: standard deviation.

### Estimated cardiorespiratory fitness and the risk of aortic valve replacement surgery

**Figure 1:**
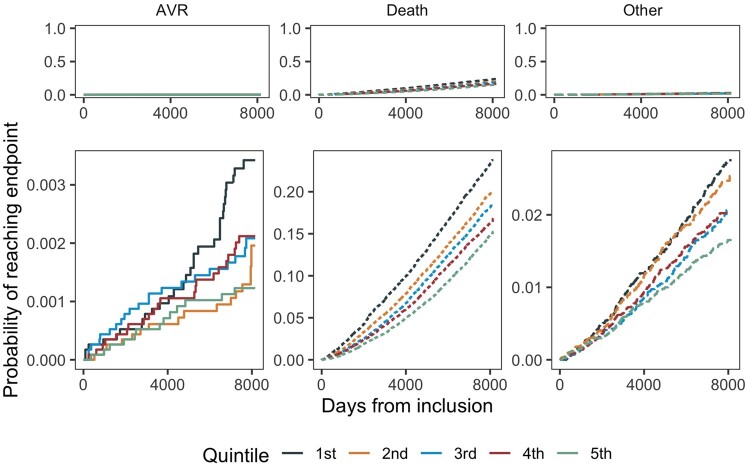
Probability of reaching end point of isolated aortic valve replacement surgery.

**Figure 2:**
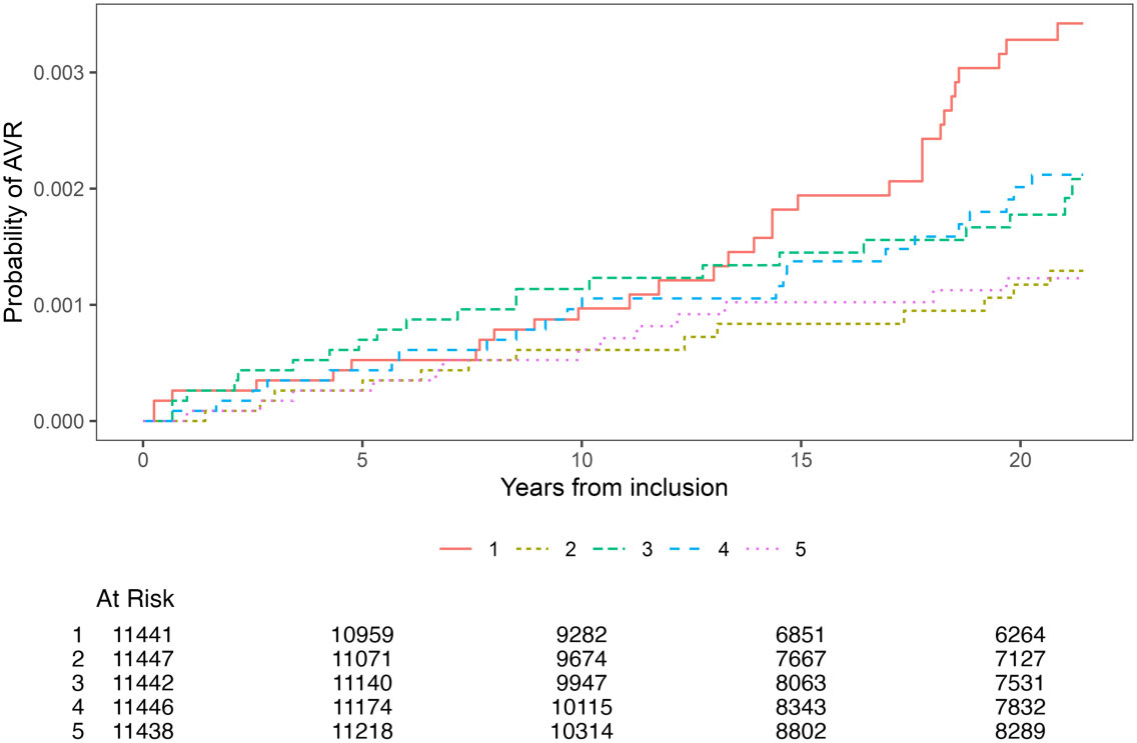
Probability of reaching end point of isolated aortic valve replacement surgery per estimated cardiorespiratory fitness quintiles.

**Figure 3:**
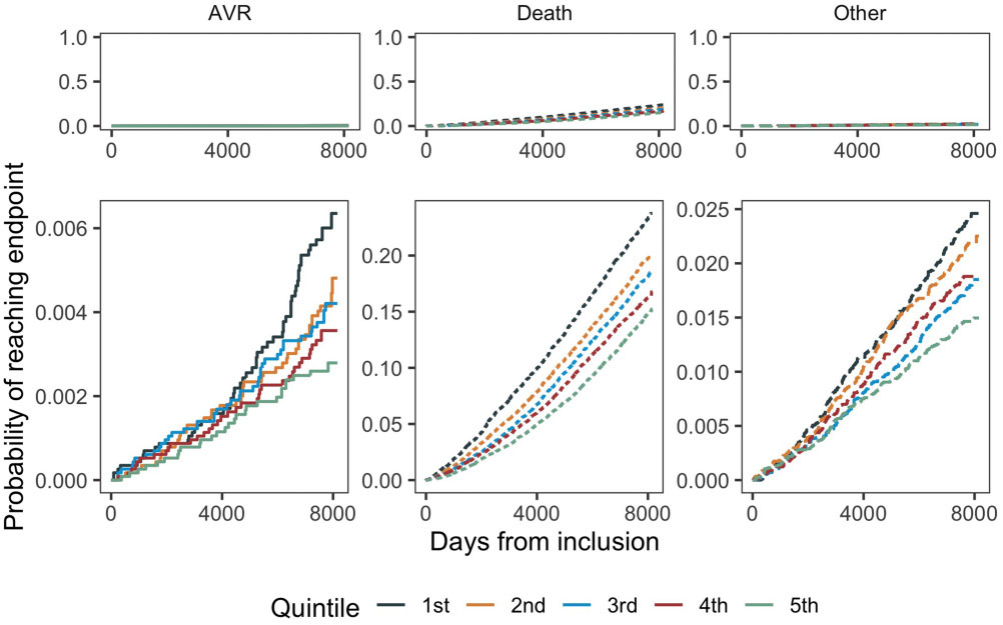
Probability of reaching end point of aortic valve replacement surgery ± concomitant procedures.

**Figure 4:**
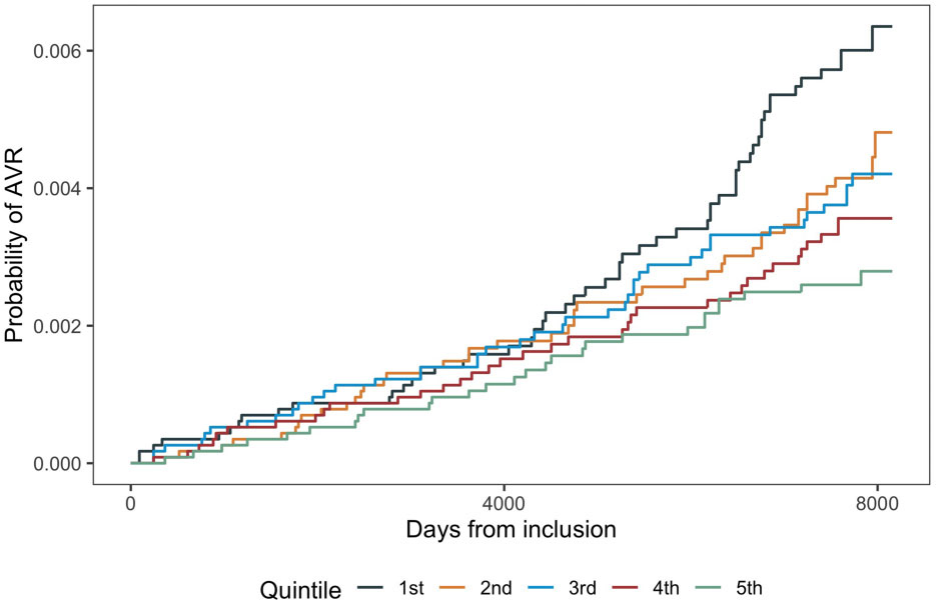
Probability of reaching end point of aortic valve replacement surgery ± concomitant procedures per estimated cardiorespiratory fitness quintile.

We found an inverse relationship between eCRF and the incidence of AVR surgery, with a 15% (95% CI 1–27) lower risk of AVR per 1-MET higher eCRF (Table 2). Participants keeping out of the lowest quintile (Q1) seemed to have a substantial lower risk of AVR surgery, with a 56% (95% CI 14–77) lower risk in the highest quintile (Q5) compared to Q1 (Figure 1 and Figure 2). Using a restricted cubic spline for the eCRF measure in the best-fit model with 4 knots (*P* < 0.001 compared to the linear per 1-MET model) showed a similar pattern as demonstrated in the analyses of the quintiles (Supplementary Material, Fig. S1). Sensitivity analyses with inclusion of all AVR surgeries due to AVS including concomitant procedures showed similar associations between eCRF and AVR surgery (Figure 3 and Figure 4), with a 14% (95% CI 5–22) lower risk of AVR surgery per 1-MET increment of eCRF (Table 3). We found a 1% (95% CI 0–2) higher risk of AVR per 1 beat higher rHR and a 2% (95% CI 0–3) higher risk per 1 cm higher waist circumference.

**Table 2:** Hazard ratios for incident isolated aortic valve replacement surgery per physical activity groups, per 1-metabolic equivalent task estimated cardiorespiratory fitness and by quintiles of estimated cardiorespiratory fitness

			Model 1		Model 2	
	*n*	Events (*n*)	HR	95% CI	HR	95% CI
Physical activity						
Less active	16 726	39	1	–	1	–
Moderate active	27 530	40	0.85	0.54–1.35	0.89	0.56–1.4
Highly active	7716	13	1.33	0.7–2.53	1.38	0.72–2.62
eCRF						
Per 1 MET	57 214	102	**0**.**85**	**0**.**73–0**.**99**	**0**.**85**	**0**.**73–0**.**99**
Q1	11 441	31	1	–	1	–
Q2	11 447	15	**0**.**48**	**0**.**26–0**.**89**	**0**.**48**	**0**.**26–0**.**89**
Q3	11 442	21	0.67	0.39–1.17	0.68	0.39–1.19
Q4	11 446	22	0.71	0.41–1.22	0.72	0.42–1.24
Q5	11 438	13	**0**.**43**	**0**.**22–0**.**84**	**0**.**44**	**0**.**23–0**.**86**

Model 1: adjusted for sex and age at inclusion. Model 2: model 1 + alcohol use, smoking status, family history of CAD, occupational status upon HUNT inclusion. event: isolated AVR, mean gradient ≥40 mmHg. Bold denotes significant results.

AVR: aortic valve replacement; CI: confidence interval; eCRF: estimated cardiorespiratory fitness; HR: Hazard ratio; HUNT: Trøndelag Health Study.

**Table 3:** Hazard ratios for incident aortic valve replacement including concomitant procedures per physical activity groups, per 1-metabolic equivalent task estimated cardiorespiratory fitness and across estimated cardiorespiratory fitness quintiles

			Model 1		Model 2	
	*n*	Events (*n*)	HR	95% CI	HR	95% CI
Physical activity						
Less active	16 726	72	1		1	
Moderate active	27 530	82	0.92	0.67–1.27	0.95	0.68–1.32
Highly active	7716	29	1.42	0.91–2.22	1.45	0.93–2.28
eCRF						
Per 1 MET	57 214	203	**0**.**85**	**0**.**78–0**.**94**	**0**.**86**	**0**.**78–0**.**95**
Q1	11 441	55	1	–	1	–
Q2	11 447	43	0.77	0.52–1.15	0.77	0.52–1.15
Q3	11 442	41	0.73	0.49–1.1	0.74	0.49–1.11
Q4	11 446	36	0.64	0.42–0.98	0.65	0.43–1.0
Q5	11 438	28	**0**.**51**	**0**.**32–0**.**8**	**0**.**52**	**0**.**33–0**.**82**

Model 1: adjusted for sex and age at inclusion. Model 2: model 1 + alcohol use, smoking status, family history of CAD, occupational status upon HUNT inclusion. Event: isolated AVR, mean gradient ≥40 mmHg. Bold denotes significant results.

AVR: aortic valve replacement; CI: confidence interval; eCRF: estimated cardiorespiratory fitness; HR: Hazard ratio; HUNT: Trøndelag Health Study.

### Physical activity and the risk of aortic valve replacement surgery

There was no statistically significant association between PA and the risk of undergoing isolated AVR (Table 2).

### Physical activity and mortality after aortic valve replacement surgery

We did not find any statistically significant associations between PA and mortality after surgery (Table 4).

**Table 4:** Hazard ratios for overall survival after isolated aortic valve replacement surgery per physical activity group, per 1-metabolic equivalent task estimated cardiorespiratory fitness and by quintiles of estimated cardiorespiratory fitness

			Model 1		Model 2	
	*n*	Events (*n*)	HR	95% CI	HR	95% CI
Physical activity						
Less active	39	16	1	–	1	–
Moderate active	40	14	0.83	0.40–1.74	0.82	0.38–1.78
Highly active	13	5	0.70	0.23–2.14	0.74	0.20–2.79
eCRF						
Per 1 MET			**0.65**	**0.51–0.83**	**0.63**	**0.47–0.83**
Q1	31	16	1	–	1	–
Q2	15	5	0.84	0.30–2.41	0.69	0.22–2.11
Q3	21	7	0.39	0.15–1.02	0.40	0.13–1.20
Q4	22	9	0.53	0.22 –1.28	0.46	0.15–1.39
Q5	13	1	**0.07**	**0.01–0.55**	**0.06**	**0.01–0.47**

Model 1: adjusted for sex, time from inclusion to surgery and age at inclusion. Model 2: model 1 + alcohol use, smoking status, family history of CAD, occupational status upon HUNT inclusion. Event: isolated AVR, mean gradient ≥40 mmHg. Bold denotes significant results.

AVR: aortic valve replacement; CI: confidence interval; eCRF: estimated cardiorespiratory fitness; HR: Hazard ratio; HUNT: Trøndelag Health Study.

### Estimated cardiorespiratory fitness and mortality after aortic valve replacement surgery

Results showed a significant 37% (95% CI 17–53) reduced risk of mortality per 1-MET higher eCRF. There was no evidence of non-linearity when testing models with restricted cubic spline terms of eCRF (best model; *P* = 0.17). When dividing patients into quintiles, there was a substantial lower risk of mortality in those in Q5 compared to Q1, but there were few patients in each quintile and results should be interpreted with caution (Table 4).

## DISCUSSION

Our main finding is that higher eCRF is associated with lower incidence of undergoing isolated AVR surgery due to AVS, with a substantial risk reduction per 1-MET higher eCRF. However, higher levels of PA were not significantly associated with reduced risk of AVR surgery. Further, higher eCRF early in life was associated with lower mortality after AVR surgery.

The results of this study are in line with a previous assessment of eCRF and incident isolated CABG surgery, where we found an 11% lower risk of CABG per 1-MET higher eCRF [11]. There are continuous efforts to establish the risk factors for degenerative valvular heart disease, and to chart how risk factors and pathophysiological mechanisms overlap with CAD [4, 6]. A large meta-analysis of observational studies showed that the presence of AVS was associated with significant increased risk of coronary events and cardiovascular mortality [19]. Calcified AVS, which is the most common aetiology of AVS, is multifactorial and involves extracellular matrix degradation, fibrosis, cellular inflammation, lipid accumulation, calcium nodule formation and neo-angiogenesis among other things [20]. As opposed to PA and exercise, which denotes behaviour, CRF is a physical attribute influenced by PA level and intensity, sex, age, genotypes, and other factors. Our results are unable to pinpoint which factors are responsible for the associated risk reduction in our population, or which pathophysiological mechanisms they affect. It is also not known whether low CRF itself is a risk factor, or if it works through improving other risk factors of AVS. However, correction for various factors from Models 1 to 2 did not alter results considerably. We postulate that eCRF potentially could be used to evaluate future risk of AVR. This means considering eCRF as a predictor of cardiovascular risk more than simply a measure of maximal oxygen uptake. Still, the low absolute risk of AVR should be kept in mind.

Our results did not imply that self-reported PA *per se* reduced the incidence of AVR surgery. A few other studies have previously investigated the associations between PA and AVS, such as Sarajlic *et al.* [21], who reported a relatively strong, but non-significant trend towards increased risk of AVS with higher PA volumes. Also, a study from Eveborn *et al.* [22] who followed 3243 participants for 14 years with repeated echocardiographic examinations and found a trend towards higher AVS risk in the most active group. Moreover, some studies on PA and CAD, which also is a calcific degenerative disease, argue that high levels of PA could accelerate coronary artery calcification [23, 24]. However, as engagement in PA is the main way to increase eCRF, one would expect to see a similar trend between PA and eCRF. Also, PA has been postulated to reduce CAD by modifying inflammation, blood pressure and endothelial dysfunction [9], which are risk factors expected to have important roles also in AVS [22, 25]. This could therefore imply that there are other factors influencing both eCRF and the development of aortic valve disease that are more prominent than PA. Exercise and PA have several haemodynamic effects on the heart, but how the heart valves are affected, is not fully understood. Moreover, it is not known how the exercise-induced benefits in the heart affect progression of valvular disease. As regular exercise increases cardiac output through increased stroke volume, one could hypothesize that this mechanical force prohibits progression of AVS. On the other hand, increased stroke volume may induce a murmur, and high levels of PA may demask symptoms at an earlier stage, leading to earlier medical examination and subsequent surgery. Some have also speculated that increased share-stress aggravates valvular calcification, as emerging evidence indicates in CAD [26]. However, our results were unable to find evidence to support these hypotheses as results were not significant and CIs were broad. Moreover, we were only able to identify patients engaging >3 h of hard PA per week and not those who engage more extreme levels of PA. To gain more insight into PA and AVS, more studies are needed, especially on individuals engaging large volumes of high-intensity exercise.

Lastly, we found that high eCRF prior to AVR was associated with a lower risk of overall mortality after AVR surgery. These results are also in line with our study on eCRF and isolated CABG surgery, where we found a 15% lower mortality risk per 1-MET higher eCRF [11]. In a large meta-analysis of 37 studies of 2 258 029 participants, authors found a 11% lower mortality risk per 1-MET higher CRF, similar to our results [27]. CRF has further been shown to be associated with mortality across the spectra of age, sex and race [28]. Moreover, CRF has been shown to forecast the short-term results after several types of surgery such as for instance short-term morbidity and mortality after CABG surgery [29]. One potential explanation for reduced mortality after surgery in our study could be a lower risk of postoperative complications. In a recent study, authors found that those undergoing AVR had decreased survival compared to the general population and that this was explained by an increased relative risk of cardiovascular death [30]. It is possible that the increased survival after AVR surgery in our study could be a result of lower cardiovascular death. However, as we had no data on the specific causes of death, and the fact that we combine short- and long-term mortality, conclusions should be drawn carefully. However, we argue that CRF could be useful to forecast survival after AVR surgery.

### Strengths and limitations

The main strengths of this study are the prospective design and the long follow-up period of a large population cohort with high participation rates. Emigration rates are known to be low in the HUNT, which reduces the chance of participants undergoing AVR surgery elsewhere. A contingent misclassification of outcomes would however be expected to be non-differential and likely give an underestimation of the effect estimates. The NCoDR is a nationwide database with information regarding all individuals who die in Norway or live in Norway and die abroad. Classification of deaths during follow-up is therefore considered complete.

Self-reporting PA inherits the risks of misreporting and cognitive limitations related to comprehension or recall. Moreover, as PA and exercise is socially desirable, people often tend to report higher levels than the actual true amounts. Validation studies of the self-reported PA questionnaire have concluded that questions regarding ‘hard activity’ have a significant correlation with VO_2_max and is considered to be a valid measure of vigorous activity [14]. The ‘light activity’ questions have some weaknesses, such as for instance that people may have difficulty remembering volumes of such activities that are to a lesser degree planned. Further, as in all observational studies, we cannot exclude the possibility of reverse confounding in our analyses despite adjustments for several important covariates. We were also unable to identify those engaging very high levels of PA, as the wording of the questions in the HUNT questionnaires only made us able to classify those with hard PA >3 h/week. Identifying those who engage excessive amount of exercise would have been of great interest.

We aimed to exclude participants who had known heart disease at baseline, but we were not able to exclude those with a subclinical AVS. It is therefore possible, and likely, that some of the study participants already had an evolving AVS upon inclusion. However, when looking at the cumulative incidence curve, the groups do not separate until some time has passed. This fits with the pathophysiology, as risk factors are present several years before symptomatic AVS.

TAVI procedures were not included as they were not systematically included in the heart surgery database throughout the study period. Further, TAVI was only a treatment option for a limited fraction of our study period and only reserved for those deemed inoperable. This could of course influence the incidence of open AVR surgery.

## CONCLUSIONS

The main finding of this study is that eCRF was strongly and inversely associated with the risk of developing AVS-demanding AVR through open-heart surgery. Having higher eCRF prior to surgery was associated with better survival after surgery. More studies are warranted to explore the utility of eCRF as a risk stratification tool both in the healthy population and in those undergoing AVR.

## Supplementary Material

ezad322_Supplementary_Data

## Data Availability

The data underlying this article were provided by the local heart surgery registry of St. Olavs hospital and from the NCoDR under licence. Data can be shared on request to the corresponding author after gaining permission from the 2 registers. Data from the HUNT cannot be shared publicly due to the privacy of the study participants, but data can be shared on reasonable request to the HUNT research centre (data access—HUNT—Faculty of Medicine—NTNU—NTNU).

## Notes

Presented at the EACTS Annual Meeting, Milan, Italy, 6 October 2022.

## ABBREVIATIONS

AVR: Aortic valve replacement
AVS: Aortic valve stenosis
CABG: Coronary artery bypass grafting
CAD: Coronary artery disease
CIs: Confidence intervals
CRF: Cardiorespiratory fitness
eCRF: Estimated cardiorespiratory fitness
HUNT: Trøndelag Health Study
MET: Metabolic equivalent of task
NCoDR: Norwegian Cause of Death Registry
PA: Physical activity
rHR: Resting heart rate
SDs: Standard deviations
TAVI: Transcatheter aortic valve implantation
