# Supplementary material for: Cardiorespiratory fitness and the incidence of surgery for aortic valve stenosis—the HUNT study
Source: Eur J Cardiothorac Surg. 2023 Sep 19;64(5):ezad322. doi: 10.1093/ejcts/ezad322 (PMC10634520; doi:10.1093/ejcts/ezad322)
Supplement: ezad322_Supplementary_Data [file ezad322_supplementary_data.pdf]

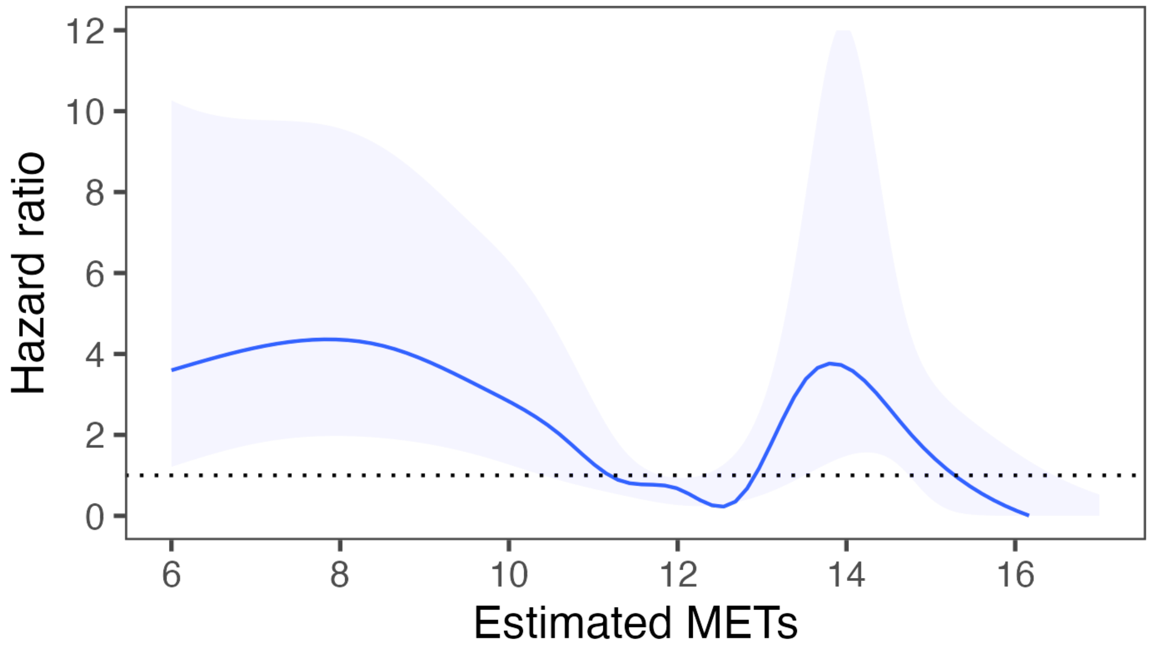

Supplemental Figure 1: Hazard ratio for isolated AVR surgery with 95% confidence intervals using restricted cubic splines
